# Isolation, identification and utilization of lactic acid bacteria from silage in a warm and humid climate area

**DOI:** 10.1038/s41598-021-92034-0

**Published:** 2021-06-15

**Authors:** Chao Peng, Wentao Sun, Xiang Dong, Lili Zhao, Jun Hao

**Affiliations:** Department of Grassland Science, Collage of Animal Science, Guizhou University, Jiaxiu South Road, Guiyang, 550025 Guizhou China

**Keywords:** Microbiology, Molecular biology

## Abstract

The study aimed to isolate and identify lactic acid bacteria (LAB) from silages and their application to improve the fermentation quality of alfalfa. Forty-nine LAB strains were isolated from silages, and two strains were screened for growth and acid production rates. Then two strains were selected for Physiological and morphological tests and 16S rRNA sequencing. They were Gram-positive and Catalase-negative and were able to grow at pH 3.5 and at 45 °C, were unable to grow different NaCl concentrations as 3.0% and 6.5%. Strain BDy3-10 was identified as *Lactobacillus rhamnosus*, while TSy1-3 was identified as *L. buchneri*. The selected strains were evaluated on fermentation of alfalfa silage. The highest crude protein content occurred in the BDy3-10 treatment group. The contents of neutral detergent fiber and acid detergent fiber in the TSy1-3 treatment were significantly lower than other treatment (*P* < 0.05). Compared to the control treatment, inoculation treatments deceased pH during ensiling (*P* < 0.001) and provided the most increased lactic acid content after ensiling for 10 days (*P* < 0.001). The acetic acid contents of all the inoculation groups were significantly increased (*P* < 0.001) during ensiling, and were lower than that of control group (*P* < 0.001). So, the TSy1-3 treatment most effectively improved the fermentation quality of alfalfa silage in warm and humid climate area.

## Introduction

Lactic acid bacteria (LAB) can ferment and produce abundant lactic acid, which is used as a silage additive. Inoculation with LAB could increase the content of lactic acid, decrease the pH^1^, and help improve the silage fermentation profile and enhance feed quality^2,3^. Previous studies have shown that LAB inoculation in silages can reduce dry matter losses and increase aerobic stability, degradability rate and animal performance^4,5^. Recently, dual-purpose inoculants containing homo-fermentative and hetero-fermentative bacteria have been developed to overcome the limitations of inoculants containing either type of bacteria alone, and the combination of both types of organisms can improve the speed of fermentation and enhance aerobic stability^6–8^. Drouin et al. reported that inoculation with a combination of additives effectively improved the fermentation quality and aerobic stability of silage^9^. Homofermentative LAB fermentation produced low levels of volatile fatty acids that could not effectively inhibit the growth of molds and yeasts, resulting in silage corruption^2^. However, heterofermentative LAB could produce high levels of acetic acid that inhibited the growth of fungi and increased the aerobic stability of silage^2^. Several studies have suggested that mixed LAB inoculants can improve aerobic stability^6,10,11^.

In recent years, increasing work has focused on how to improve fermentation quality by isolating LAB ideally capable of dominating lactic fermentation from forage or silage^12,13^. Wang et al*.* discovered that a LAB strain isolated from *Leymus chinensis* silage could be useful for promoting favourable fermentation of *Moringa oleifera* leaf silage^14^. Ennahar et al. found that the presence of *L. plantarum* dominates in rice silage^15^, while Ni et al. shown that the presence of *Ped. pentosaceus* dominates^16^. This might be due to their different environments. LAB strains isolated from different forages in different environments usually have different effects on silage fermentation^17^. Thus, it is necessary to screen local lactic acid bacteria resources suitable for the area.

Therefore, the goals of the present study were to isolate, screen and identify the LAB from superior silage in the warm and humid climate of Guizhou karst areas. After that, the evaluated excellent LAB strains were used to inoculate alfalfa silage to determine their effect on the fermentation quality.

## Materials and methods

### Silage materials and isolation of LAB

**Figure 1:**
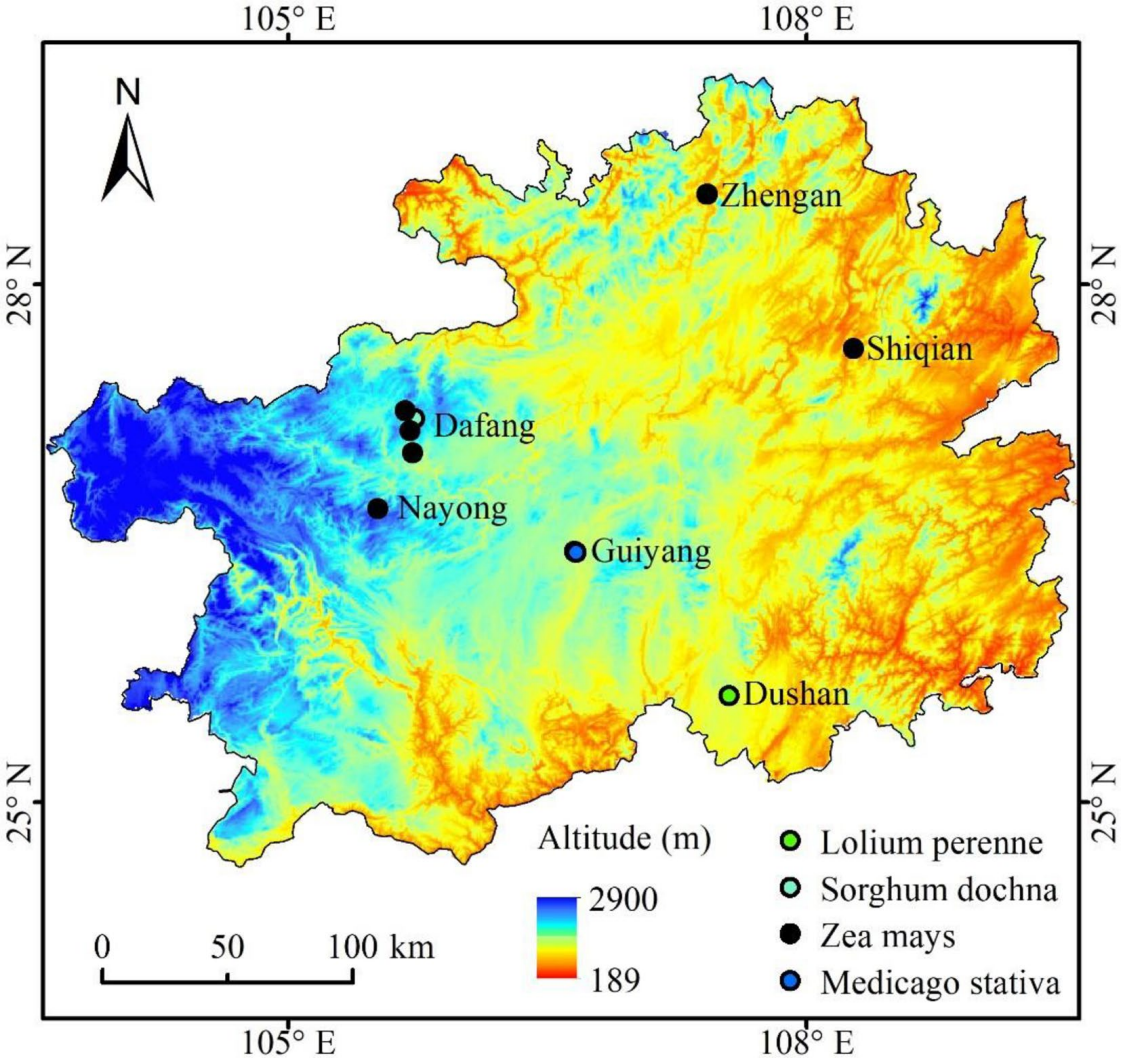
The silage materials, longitude, latitude and altitude of sampling sites. *Note* The map was generated by Arcmap 10.8 software (https://links.esri.com/open-source-acknowledgments).

12 silage raw materials that included *Lolium perenne*, *Sorghum dochna*, *Zea mays* and *Medicago stative* L., were harvested from different sites in Guizhou Province, as shown in Fig. 1 and Table 1. The study comply with the regulation of Agricultural and Rural Bureau of Guizhou for plant research. Also, the samples of plant collected in this experiment were approved by the same department. The silage raw material was chopped to about (1–2) cm using a paper-cutter, the chopped forages were packed into polyethylene plastic bags (dimensions 16 × 25 cm; Embossed Food saver bag; Taizou Wenbwu Soft-Packing Color-Printing Co. Ltd, Zhejiang, China), and approximately 600 g of forage was packed in each polyethylene bag and then vacuum-sealed. The bags were stored at room temperature for 60 d. After the silage was finished, the colour was yellow-green, the sample presented acidity and aroma, and the stem and leaf structure could be clearly identified.

**Table 1:** Annual precipitation and annual mean temperature of sampling sites (data in 2014–2018).

Sampling sites	Huaxi	Nayong	Shiqian	Zhengan	Dafang	Dushan
Annual precipitation (mm)	1268.66	1278.92	1216.36	1144.14	1169.18	1413.54
Annual mean temperature (°C)	15.84	14.54	17.86	16.36	12.8	15.92

Approximately 10 g of silages were blended with 90 ml of sterilized saline solution (8.50 g L^−1^ NaCl) and serially diluted from 10^–1^ to 10^–6^ in sterilized water. Then, the three dilutions 10^–1^, 10^–3^ and 10^–5^ were taken and coated on LAB medium plates for cultivation. By observing the appearance and morphology of colonies on the solid medium of *Lactobacillus*, a single colony with different appearances was selected, and each colony was separated and purified twice. The purified colonies were cultured on the solid medium of *Lactobacillus* under a constant temperature 37 °C and anaerobic conditions for 24 h, and then identified by Gram's staining and catalase activity. Finally, the identified *Lactobacillus* was added to the liquid nutrient medium containing sterile glycerin and preserved at − 80 °C.

### Screening of LAB

Isolation strains were initially screened due to their high acidification ability and growth efficiency at 37 °C^18^. Gram staining, colony morphology and catalase activity of screening strains were evaluated^19^. Strains were inoculated in MRS broth with different NaCl (3.0% and 6.5%) to test salt tolerance. Growth in MRS broth at pH 3, 3.5, 4, 4.5, 5, 5.5, 6, 7 and 7.5 and at temperatures 20 °C, 30 °C, 40 °C, 45 °C and 50 °C were tested.

### 16S rRNA sequencing analysis

A TSINGKE DNA kit (general type) was used to extract the 16S rRNA of screened strains. The 16S rRNA universal amplification primers were: 27F (5, -AGAGTTTGATCCTGGCTCAG-3) and 1492R (5, -GGTTACCTTGTTACGACTT-3)^20^. Then, the PCR amplification products were sent to Beijing for sequencing at Biotechnology Co., Ltd., Kunming Branch.

After obtaining 16S rRNA sequences, they were compared with the 16S rRNA sequences of type strains in GenBank. The CLUSTALW program was used to assemble and align the sequences of selected strains and typical strains.

### Silage and ensiling

Alfalfa (*Medicago sativa* L.) at early budding was harvested from Guizhou University West Campus experimental site (Guiyang, Guizhou, China) on January 1, 2019, and wilted for 48 h. The chemical and microbial compositions after wilting are shown in Table 2.

**Table 2:** Chemical and microbial compositions after alfalfa wilting.

Items	Content
DM (%)	35.72 ± 2.59
CP (% DM)	23.16 ± 1.99
NDF (% DM)	37.97 ± 1.82
ADF (% DM)	24.83 ± 1.46
WSC (% DM)	7.51 ± 0.37
CF (% DM)	29.64 ± 1.21
Ash (% DM)	11.15 ± 0.83
EE (% DM)	9.93 ± 0.82
LAB (log cfu g^−1^ FM)	3.01 ± 0.56

*DM* dry matter, *CP* crud protein, *NDF* neutral detergent fiber, *ADF* acid detergent fiber, *WSC* water soluble carbohydrates, *CF* crud fiber, *Ash* crud ash, *EE* ether extract, *LAB* lactic acid bacteria, *cfu* colony-forming units, *FM* fresh matter.

Two selected strains (BDy3-10 and TSy1-3) were used as inoculants for silage preparation. Each strain was dissolved to approximately 10^6^ colony-forming units (cfu) g^−1^ FM, and 100 mL of inoculant was dissolved and sprayed on 3.2 kg of chopped forage (1–2 cm), which was then mixed thoroughly. The chopped forage was then treated with the same amount of (1) distilled water (control), (2) *L. rhamnosus* (BDy3-10), (3) *L. buchneri* (TSy1-3), or (4) BDy3-10 + TSy1-3 at a ratio of 1:1. All the treated forages were packed into polyethylene plastic bags (dimensions 16 × 25 cm; Embossed Food saver bag; Taizou Wenbwu Soft-Packing Color-Printing Co. Ltd, Zhejiang, China), and approximately 200 g of wilted forage was packed in each polyethylene bag and then vacuum-sealed, with three replicates for each treatment. The bags were stored at room temperature and opened after 1, 6, 10, 20 and 40 days of storage, their chemical composition, fermentation quality and aerobic stability were analyzed.

### Chemical and fermentation analysis

The dry matter (DM) contents of fresh and ensiled forages were determined by drying the sample in a forced-air oven at 65 °C for 48 h. The dried samples were ground to pass a 1 mm screen by a laboratory knife mill (FW100, Taisite Instrument Co., Ltd., Tianjin, China). Crude protein (CP) was analyzed using a Kjeldahl nitrogen analyzer (Kjeltec 2300 Auto-Analyzer, FOSS Analytical AB, Hoganas, Sweden) and crude fat (EE) was determined by an extraction method^21^. Crude ash content (Ash) was detected in an ash furnace by burning at 550 °C for 4 h. Crude fiber (CF), neutral detergent fiber (NDF) and acid detergent fiber (ADF) contents were measured by an A220 Fiber Analyzer (ANKOM Technology Corp., Macedon, NY, USA)^22^. Water soluble carbohydrate (WSC) was determined using the thracenone-sulphuric acid method^23^.

Twenty grams of each silage sample was mixed with 180 mL of distilled water, stored at 4 °C for 18 h, and then filtered. The pH of this filtrate was measured by a glass electrode pH meter (PHS-3C, INESA Scientific Instrument Co., Ltd, Shanghai, China), and ammonia-N was determined by steam distillation of the filtrates. The concentration of organic acids (lactic acid, acetic acid, propionic acid and butyric acid) was measured using high performance liquid chromatography (column, Shodex RSpak KC-811S-DVB gel C; 8.0 mm × 30 cm; Shimadzu, Tokyo, Japan); oven temperature, 50 °C; mobile phase, 3 mmol/L HClO4; flow rate, 1.0 mL/min; injection volume, 5 μL; and a SPD-M10AVP detector^24^.

### Statistical analysis

The 16S rRNA sequences of the LAB isolates were analyzed by MEGA 6.0 for Windows (The Biodesign Institute, Tempe, AZ). Statistical analyses of data for chemical composition, fermentation characteristics and aerobic stability were performed using one-way ANOVA of the Statistical Package for Social Sciences (SPSS Version 19.0, SPSS Inc., Chicago, IL, USA). Turkey’s honest significant difference (HSD) test was employed for different sample means and the significance was declared at *P* < 0.05. Sigma Plot 10.0 is plotted.

## Results

### Screening of LAB

As shown in Table 3, forty-nine strains of LAB were isolated from silages. We initially screened two strains by their high growth and acid-producing rates for 24 h at 37 °C, the OD value of strain TSy1-3 at 24 h was significantly largest that other strains (*P* < 0.05), at 3.02. The lowest pH value occurred in the BDy3-10 treatment group (*P* < 0.05), at 3.75.

**Table 3:** OD and pH values of lactic acid bacteria cultured in MRS medium for 24 h.

Strain number	OD	pH	Strain number	OD	pH
TSy1-3	3.02 ± 0.05a	3.94 ± 0.07jk	ZZt1-20	1.88 ± 0.09ijkl	4.14 ± 0.11gh
BDy3-10	2.75 ± 0.04b	3.75 ± 0.04q	BDy2-29	1.83 ± 0.06jklm	4.16 ± 0.24g
BDy3-41	2.69 ± 0.14b	4.06 ± 0.05g	ZZt1-6	1.80 ± 0.10klm	4.08 ± 0.03gh
TSy1-10	2.63 ± 0.09bcd	3.78 ± 0.04p	BDy2-19–1	1.80 ± 0.07lm	4.60 ± 0.10ef
BDy3-7	2.63 ± 0.07bcd	3.86 ± 0.03m	TSy1-6	1.74 ± 0.09lmn	4.49 ± 0.06f
ZZt1-14	2.62 ± 0.04bcd	3.88 ± 0.11lm	BDy3-16-2	1.73 ± 0.16lmn	4.01 ± 0.00gh
TSy1-45	2.62 ± 0.12bcd	4.02 ± 0.01gh	BDy2-50	1.71 ± 0.10mn	4.07 ± 0.05gh
BDy3-43	2.54 ± 0.07cd	4.09 ± 0.07gh	BDy3-40	1.71 ± 0.10mn	4.69 ± 0.16de
GHy1-2	2.53 ± 0.06d	3.97 ± 0.01hi	BDy3-14	1.69 ± 0.14mn	4.76 ± 0.17e
BNy1-12	2.53 ± 0.05d	3.85 ± 0.12mn	BNy1-1	1.69 ± 0.10mn	4.93 ± 0.06bc
BDy3-35	2.36 ± 0.10e	3.78 ± 0.04p	BNy1-16	1.68 ± 0.10mn	4.82 ± 0.19d
TSy1-46	2.35 ± 0.09e	4.00 ± 0.04h	BDy2-47	1.63 ± 0.06no	4.73 ± 0.13d
BNy1-8	2.30 ± 0.09ef	4.03 ± 0.07gh	BDy2-62	1.61 ± 0.09no	5.00 ± 0.07b
ZZt1-16	2.26 ± 0.08ef	4.01 ± 0.02gh	GHy1-10	1.58 ± 0.06no	5.00 ± 0.01b
BDy3-39	2.25 ± 0.03ef	3.86 ± 0.11m	GHy1-1	1.52 ± 0.09o	5.03 ± 0.04b
BDy2-46	2.15 ± 0.15fg	4.04 ± 0.03gh	TSy1-34-1	1.51 ± 0.08o	3.83 ± 0.09o
BNy1-2	2.14 ± 0.04fg	4.10 ± 0.08gh	TSy1-39	1.49 ± 0.04op	4.50 ± 0.01f
TSy1-33	2.06 ± 0.12h	3.92 ± 0.10kl	GHy1-8	1.47 ± 0.10op	4.98 ± 0.05b
GHy1-11	2.06 ± 0.06h	4.06 ± 0.04gh	TSy1-1	1.36 ± 0.08pq	5.07 ± 0.06b
ZZt1-18	2.06 ± 0.08h	4.05 ± 0.12gh	BDy2-35	1.27 ± 0.01q	5.08 ± 0.04b
ZZt1-22	2.02 ± 0.14hi	3.95 ± 0.03i	BNy1-7	1.25 ± 0.09q	5.09 ± 0.08b
ZZt1-3	1.97 ± 0.05hij	4.13 ± 0.16gh	TSy1-30	1.22 ± 0.05q	3.77 ± 0.04p
ZZt1-5	1.97 ± 0.11hijk	4.09 ± 0.15gh	BNy1-4	1.12 ± 0.08r	5.55 ± 0.10a
ZZt1-7	1.95 ± 0.10hijkl	4.12 ± 0.10gh	BNy1-6	0.82 ± 0.04s	5.56 ± 0.18a
BDy2-17	1.89 ± 0.06ijkl	4.08 ± 0.11gh			

Values with different lowercase letters show significant (*P* < 0.05).

The characteristics and type strains of screened LAB strains are shown in Table 4. Two strains were Gram-positive and Catalase-negative. Strain BDy3-10 was a homofermentative LAB, and TSy1-3 was a heterofermentative LAB. The two strains grew normally in the range of 20–45 °C, but grew weakly at 10 °C and 50 °C. They were able to grow at pH values ranging from 3.5 to 7.0, and grew weakly at pH 3.0 tolerating salt (MRS with 3.0% and 6.5% NaCl concentrations, respectively) which limited their growth.

**Table 4:** Physiological and biochemical characteristics of LAB.

Characteristics	BDy3-10	TSy1-3
Gram stain	+	+
Fermentation type	Homo	Hetero
Catalase	−	−
**Growth at pH**		
3.0	w	w
3.5	+	+
4.0	+	+
4.5	+	+
5.0	+	+
6.0	+	+
6.5	+	+
7.0	+	+
**Growth at temp**		
10 °C	w	w
20 °C	+	+
35 °C	+	+
40 °C	+	+
45 °C	+	+
50 °C	w	w
**Growth in NaCl**		
3%	−	−
6.5%	−	−

+ positive.

− negative.

*w* weakly positive, *Homo* homofermentative, *Hetero* heterofermentative.

### 16S rRNA analysis of screened LAB

The 16S rRNA sequences were compared using the NCBI database (see Table 5), and the results showed that the similarities between all sequences obtained here and several the known 16S rRNA gene sequences in the database were 99.0–100.0%.

**Table 5:** BLAST alignment of 16S rRNA sequences of LAB.

Strain number	Related species	Gene bank strain number	Similarity (%)
BDy3-10	*Lactobacillus rhamnosus*	LR134331.1	99.70
TSy1-3	*Lactobacillus buchneri*	KR055504.1	98.80

**Figure 2:**
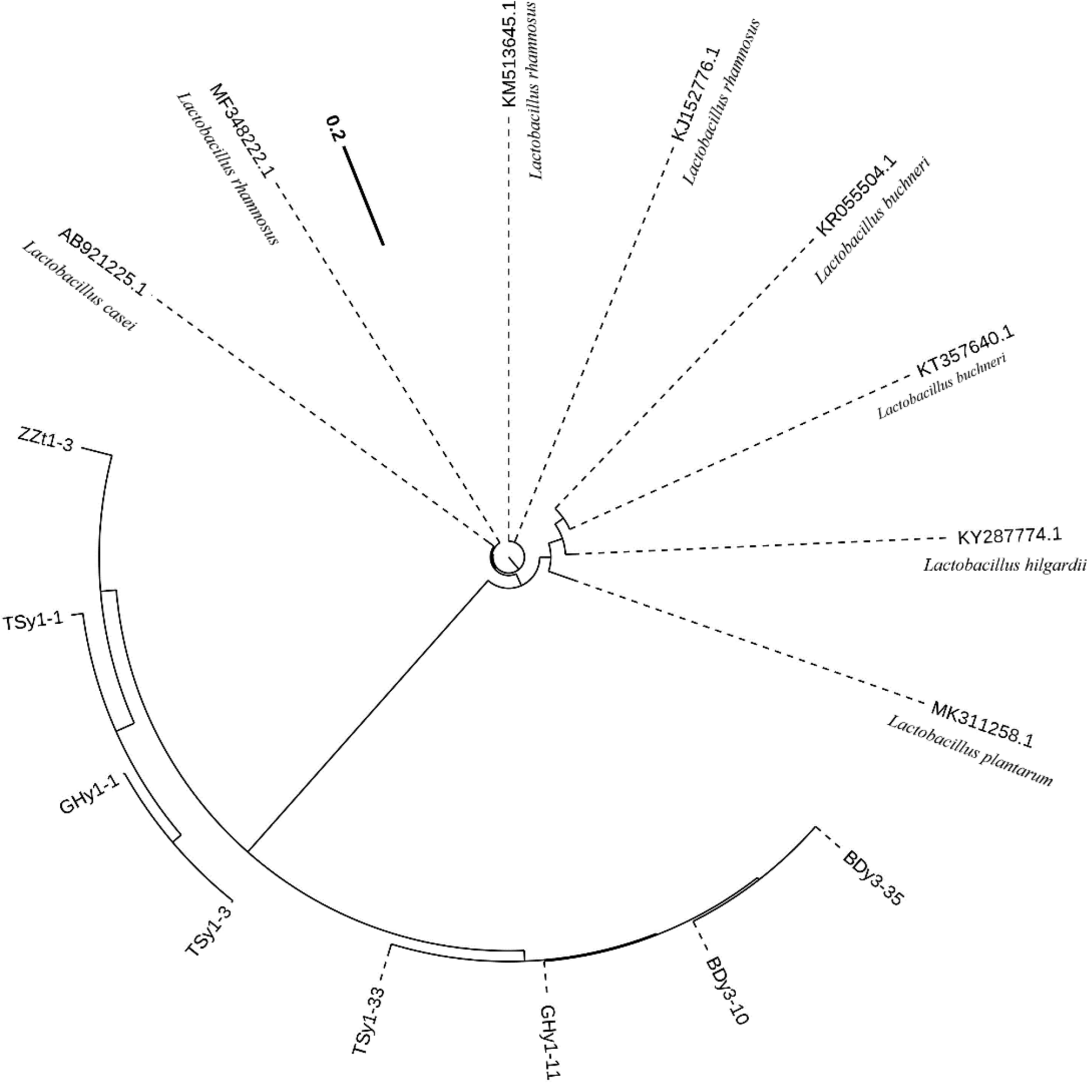
Polygenetic tree showing the relative positions of BDy3-10, TSy1-3 and related species, as inferred by the neighbor-joining method using complete 16S rRNA sequences.

The phylogenetic tree of the partial 16S rDNA sequence of BDy3-10 and TSy1-3 are presented in Fig. 2. The sequence of strain BDy3-10 is closely related to that of *L. rhamnosus*, with 99.7% similarity in their 16S rRNA gene sequences. The 16S rRNA gene sequence of the TSy1-3 strain showed 98.8% similarity to the corresponding sequence, in *L. buchneri*, confirming its identity.

### Effect of screened strains on the chemical compositions and aerobic stability of alfalfa silage

The results of the alfalfa silage chemical composition analysis are shown in Table 6. The DM and WSC contents of the TSy1-3 group treatment were significantly higher than that of the CK group (*P* > 0.05). The CP contents of all additive treatment groups were significantly higher than that of the CK group (*P* > 0.05), while the BDy3-10 treatment had the highest CP content. The CF content of the combination of TSy1-3 strain and BDy3-10 strain treatments was lower than the CK group, but the alone inoculation of TSy1-3 strain and BDy3-10 strain were higher than the CK group. The NDF and ADF contents in the TSy1-3 treatment group were significantly lower than those in the other groups (*P* < 0.05), and the EE and Ash contents in all treatments were not significantly different (*P* > 0.05). After 40 d of ensiling, the aerobic stability of all additive treatment groups was significantly higher than the CK treatment group (*P* < 0.05), and the TSy1-3 was the highest, at 173 h.

**Table 6:** Chemical composition and aerobic stability after 40 days of ensiling.

Item	CK	BDy3-10	TSy1-3	BDy3-10 + TSy1-3
DM (% FM)	33.49 ± 0.01b	32.24 ± 0.25d	34.31 ± 0.31a	33.04 ± 0.15c
CP (% DM)	20.00 ± 0.55b	21.86 ± 0.54a	21.24 ± 0.66a	21.41 ± 0.48a
EE (% DM)	9.01 ± 0.57	9.34 ± 0.24	9.26 ± 0.35	9.81 ± 1.53
CF (% DM)	26.21 ± 5.68	27.45 ± 1.19	27.16 ± 1.23	25.69 ± 1.52
NDF (% DM)	32.14 ± 0.88a	33.84 ± 0.95a	28.81 ± 1.23b	32.93 ± 1.87a
ADF (% DM)	20.26 ± 0.94a	21.14 ± 0.31a	17.90 ± 0.88b	21.14 ± 0.60a
WSC (% DM)	1.47 ± 0.07b	0.99 ± 0.02c	1.94 ± 0.05a	1.06 ± 0.03c
Ash (% DM)	10.85 ± 0.81	11.20 ± 0.55	10.61 ± 0.57	10.55 ± 0.13
Aerobic stability (h)	121 ± 0.87d	129 ± 0.59c	173 ± 0.42a	144 ± 0.43b

Values with different lowercase letters show significant (*P* < 0.05) differences in the same treatment.

*CK* control, *BDy3-10 L. rhamnosus*, *TSy1-3 L. buchneri.*

### Effects of isolated strains on the fermentation quality of alfalfa during ensiling

The changes in LA, AA and PA that occurred during ensiling are shown in Table 7. The LA content reached a maximum value for T_10_ silages, and then decreased dramatically until T_40_ in all treatments (*P* < 0.001). AA and PA contents increased with prolonged fermentation (*P* < 0.05). The content of LA was generally higher in the BDy3-10 treatment than in the other treatments during ensiling, except at T_40_ (*P* < 0.05), and reached a maximum value (57.03 mmol) for the T_10_ silage. The AA content was significantly higher in the CK silage than in the inoculated silages during ensiling (*P* < 0.05). The PA contents of all treatment groups were significantly lower than that of the CK treatment group (*P* < 0.05).

**Table 7:** Contents of LA, AA, PA and pH dynamic changes during ensiling.

Items	Ensiling day	CK	BDy3-10	TSy1-3	BDy3-10 + TSy1-3	*P* value
LA (mmol)	T_1_	1.05 ± 1.05Bb	1.70 ± 0.10Da	1.29 ± 0.38Bab	1.32 ± 0.20Cab	0.010
	T_6_	39.70 ± 0.79Ab	45.85 ± 1.07Ba	44.21 ± 1.61Aa	44.84 ± 1.11Aa	< 0.001
	T_10_	40.84 ± 3.38Ac	57.03 ± 0.09Aa	47.15 ± 1.35Ab	46.09 ± 0.75Ab	< 0.001
	T_20_	39.54 ± 6.83Ab	47.18 ± 3.26Ba	42.4 ± 0.56Aab	37.31 ± 1.29Bb	0.023
	T_40_	40.27 ± 4.23A	42.88 ± 0.35C	42.98 ± 5.53A	38.98 ± 2.67B	0.255
*P* value		< 0.001	< 0.001	< 0.001	< 0.001	
AA (mmol)	T_1_	3.61 ± 1.30Ca	1.16 ± 0.03Db	1.50 ± 0.05Eb	1.90 ± 0.04Cb	0.002
	T_6_	9.77 ± 1.11Ba	2.93 ± 0.03Cb	3.58 ± 0.14Db	3.40 ± 0.33Cb	< 0.001
	T_10_	10.95 ± 1.54ABa	6.05 ± 0.29Bb	6.56 ± 0.16Cb	7.43 ± 1.30Bb	< 0.001
	T_20_	11.45 ± 0.96ABa	7.22 ± 1.73ABc	8.59 ± 0.26Bb	8.30 ± 0.49ABbc	0.463
	T_40_	13.10 ± 0.99Aa	8.54 ± 0.22Ac	11.11 ± 0.01Ab	9.95 ± 1.78Abc	< 0.001
*P* value		< 0.001	< 0.001	< 0.001	< 0.001	
PA (mmol)	T_1_	0.14 ± 0.04	0.11 ± 0.02B	0.11 ± 0.03B	0.10 ± 0.01B	0.202
	T_6_	0.21 ± 0.03a	0.11 ± 0.05Bb	0.15 ± 0.01Bb	0.11 ± 0.01Bb	0.010
	T_10_	0.29 ± 0.05	0.33 ± 0.06A	0.29 ± 0.06A	0.34 ± 0.01A	0.624
	T_20_	0.55 ± 0.30	0.34 ± 0.05A	0.30 ± 0.06A	0.36 ± 0.01A	0.117
	T_40_	0.60 ± 0.22	0.34 ± 0.10A	0.32 ± 0.07A	0.40 ± 0.09A	0.181
*P* value		0.019	< 0.001	< 0.001	< 0.001	
pH	T_1_	6.42 ± 0.12A	6.24 ± 0.25A	6.06 ± 0.08A	6.19 ± 0.16A	0.263
	T_6_	6.26 ± 0.12Aa	5.16 ± 0.02Bb	5.03 ± 0.07Bb	5.12 ± 0.06Bb	< 0.001
	T_10_	6.24 ± 0.18ABa	5.05 ± 0.26Bb	5.10 ± 0.16Bb	5.01 ± 0.17Bb	< 0.001
	T_20_	5.98 ± 0.11Ba	5.12 ± 0.02Bb	4.99 ± 0.03Bb	5.00 ± 0.08Bb	< 0.001
	T_40_	5.58 ± 0.05Ca	5.08 ± 0.02Bb	5.00 ± 0.03Bc	5.06 ± 0.02Bbc	< 0.001
*P* value		< 0.001	< 0.001	< 0.001	< 0.001	

^a–^^c^Means having different letter superscripts within column are significantly different (*P* < 0.05).

^A–E^Means having different letter superscripts within row are significantly different (*P* < 0.05).

*LA* lactic acid, *AA* acetic acid, *PA* propionic acid.

The pH values at T_1_ ensiling time were approximately 6.0 for all treatments and decreased progressively from T_1_ to T_40_. The lowest pH value was recorded for the TSy1-3 treatment at T_20_ (4.99). The pH value of the CK treatment at T_40_ was 5.58, which was significantly higher than those of all inoculant treatments (*P* < 0.001). All inoculant treatments were significantly similar in terms of pH values at the T_40_ ensiling time, and all values tended to be 5.00.

## Discussion

In the present study, biochemical and phylogenetic analyses revealed that all of the characterized LAB belong to the genus *Lactobacillus*. However, previous studies found that the natural fermentation processes in forage crop and grass silages were dominated by *Leuconostoc*, *Lactococcus*, *Enterococcus*, and *Pediococcus*, not *Lactobacillus* species^25–27^. A plausible reason may be that the bacterial colonization of fresh crops and plants is controlled by many factors, such as the plant material. Shah et al. found^28^ that king grass silage was dominated by *P. acidilactici* and *L. plantarum*, while Italian ryegrass (*Lolium multiflorum* Lam.) silage was dominated by *P. acidilactici* and *L. rhamnosus*^12^. However, Ennahar et al. reported^15^ that the LAB species of paddy rice silage in Japan, included *P. acidilactici* and *Weissella kimchi*. In addition, Ni et al. found^16^ that the LAB species of rice silage in Henan, China include *P. pentosaceus*, *Enterococcus mundtii* and *L. garvieae*. Hence, the LAB species of the same material in different regions were also different. The LAB characteristics and storage temperature exerted strong effects on the fermentation quality^29^. The strain BDy3-10 (*L. rhamnosus*) had a high acid production rate that could result in the inhibition of aerobic microorganism activities and the reduction of fermentation substrates, while the strain TSy1-3 (*L. buchneri*) had the fastest growth, which that could shorten fermentation time, decrease nutritional loss and improved silage quality. The temperature of silage may rise to above 40 °C at the beginning of ensiling, which is due to the continuous plant respiration and activity of aerobic microorganisms when air still exists in the plant gap, particularly in the tropics and subtropics^30^. These results were consistent with those of our study, in which the screened strains BDy3-10 and TSy1-3 could grow at 45 °C, but they were both unable to tolerate salt. These results implied that they are very tolerant of acid and high temperature, satisfying the demands for growth in low-pH and high-temperature environments. This is a suitable method for silage preparation in warm and humid areas.

Previous studies reported that LAB inoculation could reduce DM loss during ensiling^31,32^. Higher residual WSC contents indicate smaller DM losses during fermentation that result in silage with a higher nutritive value^33^. In our study, lower DM losses and higher residual WSC contents occurred in the TSy1-3 treatment than in the other groups. Because the TSy1-3 strain grew fast in 24 h, it ensured rapid and vigorous LA fermentation and faster reduction of silage pH at earlier stages, which depressed the loss of WSC by fermentation via undesirable bacteria. The low NDF and ADF contents had a positive effect on the silage nutritive value and enhanced digestibility. In our study, the NDF and NDF contents in all inoculant treatments were lower than those in the control. This was similar to the result showing that inoculating LAB results in the highest decline of ADF and NDF, which improves feed intake and digestibility^34^. CP is the critical factor affecting the quality of commercial feed and roughage for ruminants^35^. Higher CP contents were obtained by all inoculants compared to the control. This could be related to the rapid reduction in pH caused by the addition of inoculants, which inhibited the growth and proteolytic activity of microorganisms such as Clostridia^36^. The highest CP content was found in the BDy3-10 treatment. BDy3-10 is a homofermentative LAB that is more efficient in lactic acid production than heterofermentative LAB and can ferment a wide variety of substrates and quickly produce large amounts of lactic acid^37^.

The pH value is considered a very important indicator for the fermentation profile and fermentation quality of ensiled materials^38^. The pH values of the control silages were generally higher than those of the inoculated silages. It is generally desired that the pH value is approximately 3.8–4.2 for any high-quality silage. In this study, the measured pH values reached approximately 5.00 at T_40_ with inoculation. This was because alfalfa is a high-protein forage crop that has a high buffering capacity and a slow rate of pH reduction during ensiling^37^. This result was similar to results from studies in which the pH value of alfalfa silage inoculated with LAB was 4.8–5.3^13,39^.

Various studies have reported that the application of homo-LAB inoculants can beneficially enhance the LA concentration and decrease AA^40^. Compared with homo-LAB fermentation, fermentation dominated by hetero-LAB is characterized by a higher pH and AA content but lower LA content^34,41^. This was consistent with inoculation results in this study. Treatment BDy3-10 had a higher LA content, and treatment TSy1-3 had a higher AA because strain BDy3-10 is a homo-LAB and strain TSy1-3 is a hetero-LAB. Furthermore, the higher AA can increase the aerobic stability of silage^8^, and it may have inhibited the growth and proteolytic activity of microorganisms such as clostridia^42^.

## Conclusion

The two strains used in this study were suitable as inoculants for alfalfa silage, and the TSy1-3 strains is good choice as an addictive in silage under the warm and humid climate in karst area.
